# Establishing Diagnostic Reference Levels for Mammography Digital Breast Tomosynthesis, Contrast Enhance, Implants, Spot Compression, Magnification and Stereotactic Biopsy in Dubai Health Sector

**DOI:** 10.3390/jimaging11030079

**Published:** 2025-03-07

**Authors:** Entesar Z. Dalah, Maryam K. Alkaabi, Nisha A. Antony, Hashim M. Al-Awadhi

**Affiliations:** 1 Central Diagnostic Imaging Department, Dubai Health, Dubai P.O. Box 2727, United Arab Emirates; 2 College of Medicine, Mohammed Bin Rashid University, Dubai Health, Dubai P.O. Box 2727, United Arab Emirates; 3 Medical Imaging Department, Dubai Hospital, Dubai Health, Dubai P.O. Box 2727, United Arab Emirates

**Keywords:** contrast-enhanced mammogram, stereotactic biopsy-guided mammogram, digital breast tomosynthesis, spot compression with magnification, implant mammogram, diagnostic reference levels (DRLs), United Arab Emirates (UAE)

## Abstract

The aim of this patient dose review is to establish a thorough diagnostic reference level (DRL) system. This entails calculating a DRL value for each possible image technique/view considered to perform a diagnostic mammogram in our practice. Diagnostic mammographies from a total of 1191 patients who underwent a diagnostic mammogram study in our designated diagnostic mammography center were collected and retrospectively analyzed. The DRL representing our health sector was set as the median of the mean glandular dose (MGD) for each possible image technique/view, including the 2D standard bilateral craniocaudal (LCC/RCC) and mediolateral oblique (LMLO/RMLO), the 2D bilateral spot compression CC and MLO (RSCC/LSCC and RSMLO/LSMLO), the 2D bilateral spot compression with magnification (RMSCC/LMSCC and RMSMLO/LMSMLO), the 3D digital breast tomosynthesis CC and MLO (RCC/LCC and RMLO/LMLO), the 2D bilateral implant CC and MLO (RIMCC/LIMCC and RIMMLO/LIMMLO), the 2D bilateral contrast enhanced CC and MLO (RCECC/LCECC and RCEMLO/LCEMLO) and the 2D bilateral stereotactic biopsy guided CC (SBRCC/SBLCC). This patient dose review revealed that the highest MGD was associated with the 2D bilateral spot compression with magnification (MSCC/MSMLO) image view. For the compressed breast thickness (CBT) group 60–69 mm, the median and 75th percentile of the MGD values obtained were MSCC: 3.35 and 3.96, MSMLO: 4.14 and 5.25 mGy respectively. Obvious MGD variations were witnessed across the different possible views even for the same CBT group. Our results are in line with the published DRLs when using same statistical quantity and CBT group.

## 1. Introduction

A diagnostic mammogram is concerned with evaluating lesions of clinical or radiological abnormality. This may require performing not only additional breast image views but also additional breast mammogram studies using different techniques. Depending on the presented clinical indication and the individual’s history, different supplementary breast image views are considered. In addition to the standard 2D digital bilateral right (R)/left (L) craniocaudal (RCC/LCC) and mediolateral oblique (RMLO/LMLO) breast image views, other breast image views are also considered, such as the following: the 2D bilateral spot compression CC and MLO, the 2D bilateral spot compression with magnification CC and MLO and the 2D bilateral implant CC and MLO [1,2]. Furthermore, the 3D digital breast tomosynthesis (DBT) technique is known for its superior breast cancer detection [3,4]. The 3D DBT technique makes use of multiple low-dose projections, allowing one to display thin axial image slices of the breast [5,6]. Amongst the additional breast studies considered for diagnosing breast abnormalities is the unilateral or bilateral contrast-enhanced mammogram (CEM). Such a study is based on the acquisition of dual-energy (high and low) mammograms that are acquired a few minutes after the injecting of iodine contrast [7]. While the low-energy acquired mammograms resemble the 2D standard bilateral CC and MLO views, high-energy acquired mammograms reveal breast tissue perfusion characteristics [8,9] and enhance the visualization of tumor neovascularity [10]. Additionally, the 2D stereotactic breast guided biopsy is considered for histological assays and the confirmation of breast tissue abnormalities [9].

Apart from the standard 2D CC and 2D MLO, every other breast image view or technique is performed to assist in accurately diagnosing a breast abnormality. The choice is usually made by a breast-imaging specialized radiologist. The breast image views/techniques are based on the patient’s history and clinical indications. For instance, a better visualization of a classification lesion is made using magnification and spot compression techniques. For benign vs. malignant identified lesions, a 3D breast tomosynthesis and contrast-enhanced techniques are needed. To confirm the diagnosis, a biopsy is needed and for augmented breasts, implant mammography views are needed. Some techniques and views require special image receptors, i.e., detectors such as for magnification and spot compression views. Others require dedicated software such as for breast tomosynthesis, and some require different imaging techniques, such the contrast-enhanced mammography implant and biopsy views. The radiological setup to perform each technique and view are out of the scope of this study. Whether a patient should undergo all possible techniques and views is determined by the radiologist and based on the patient’s clinical indications.

Given the substantial involvement of supplementary breast mammogram views and additional breast studies in the diagnosis of breast abnormalities, it is highly important to assess the radiation exposure to the breast tissue [11] and the resultant radiation-associated risks [12]. This is particularly important, especially since radiation doses vary across the different mammogram supplementary views, as reported in [13].

Today, clinicians, radiologists, scientists and technologists all have a responsibility for observing patient radiation safety [14] and implementing radiation dose management to ensure patient radiation protection [15]. To this end, diagnostic reference levels (DRLs) have been proven effective in achieving radiation dose optimization [16,17] and internal auditing by comparing Typical DRLs (TDRLs) (representing a center or facility) to Local DRLs (LDRLs) (representing several centers or a healthcare sector) or by comparing LDRLs alongside national or regional DRLs [18]. The DRL approach indirectly helps to protect individuals from been overexposed, ultimately resulting in reduced radiation-induced risks [19,20]. Both organ dose and mean glandular dose (MGD) have been accepted by the International Commission on Radiological Protection (ICRP) report 135 [18] as a dose quantity to calculate the DRL for mammography. The dose quantity, MGD, has also been accepted for predicting radiation-induced cancer risks [12,21,22].

This health sector patient dose report is the first comprehensive patient dose review conducted for diagnostic mammography in the Dubai Health sector. A comprehensive patient dose review for screening mammography that represented our healthcare sector was published by Dalah et al. [17].

This patient dose review aims to establish an inclusive DRL system to optimize radiation exposure for patients subjected to diagnostic mammograms in the Dubai Health sector. In light of this, a DRL value will be established for each possible breast image view and technique offered in our practice. This includes spot compression, spot compression with magnification, DBT, implant, contract enhancement and stereotactic biopsy breast image views. In term of mammogram study, a DRL value will be established for type of study, including digital diagnostic without contrast, digital diagnostic with contrast and digital stereotactic biopsy-guided mammograms. Moreover, the DRL values per breast view will be defined based on well-structured and categorized compressed breast thickness (CBT) groups.

This paper is organized to cover a literature review in Section 1 (introduction); the methodology is covered in Section 2 (material and methods), the study observation and findings are presented in Section 3 (results), a discussion of the findings and the limitations encountered are presented in Section 4 (discussion), and finally we state the study’s conclusions in Section 5.

## 2. Material and Methods

This retrospective patient dose health sector review was approved by our institutional scientific research ethics committee. Diagnostic mammography dose data were collected over a 9-month period, from 1 November 2023 to 1 July 2024. Diagnostic mammogram studies were provided from the one single designated center that offers a diagnostic mammography service in our healthcare sector. The center is facilitated with a digital GE Sonography Essential mammogram unit (General Electric Healthcare). A qualified medical physicist performs annual and quarterly quality assurance (QA) tests to ensure radiation exposure reproducibility.

In this diagnostic mammography health sector dose report, we collected the total dose per diagnostic mammogram study, as well as reporting the dose per each technique/view separately. Mammogram studies performed for diagnosis, in our practice, are classified into three studies that are labeled diagnostic, diagnostic with contrast and stereotactic biopsy. A diagnostic study may include all or selective image views, depending on the clinical indication and patient history. In our practice, the breast image views considered in the present diagnostic mammogram study include the following: the standard 2D LCC/RCC, LMLO/RMLO, the 2D bilateral spot compression CC and MLO (denoted as 2D RSCC/LSCC and 2D RSMLO/LSMLO), the 2D bilateral spot compression with magnification (denoted as 2D RMSCC/LMSCC and 2D RMSMLO/LMSMLO), the 3D digital breast tomosynthesis CC and MLO (denoted as 3D RCC/LCC and 3D RMLO/LMLO) and the 2D bilateral implant CC and MLO (denoted as 2D RIMCC/LIMCC and 2D RIMMLO/LIMMLO). For the contrast-enhanced diagnostic mammogram, which can be a unilateral or bilateral study, the breast image views considered include the 2D standard LCC/RCC and LMLO/RMLO and the 2D bilateral contrast enhanced CC and MLO (denoted as 2D RCECC/LCECC and 2D RCEMLO/LCEMLO). Finally, for the stereotactic biopsy-guided mammogram, the breast image views considered include the 2D standard LCC/RCC and LMLO/RMLO and the 2D stereotactic biopsy-guided CC (denoted as 2D SBRCC/SBLCC and 2D SBRMLO). Per our practice, the stereotactic biopsy-guided technique is limited to the CC view only.

The organ dose quantity measured in mGy was considered to establish this comprehensive DRL system for diagnostic mammograms. The mammography organ dose quantity for all patients enrolled in this study was automatically retrieved using the patient dedicated radiation dose tracking and management platform, DOSE TQM version 19.11 (Qaelum NV, Belgium) [23]. This dose platform is linked with the Dubai Health sector picture-archiving and communication system (PACS). Similar to our health sector screening mammography review [17], the organ dose values used here were extracted from the dose platform and DICOM tag (0040,0316). This reflects the same exact value reported by the mammography unit as MGD in mGy. Therefore, the organ dose quantity will be denoted MGD throughout this work. Patient age, CBT and mammography acquisition parameters, including filter/target material, half-value layer (HVL), peak kilovoltage (kVp), tube current–time product (mAs) and the focal spot size for each breast image view and mammogram study, were retrieved using our patient dose platform.

Given that a single center is designated for diagnostic mammography in our healthcare sector, the TDRL will represent our health sector. It can be also be considered the LDRL following ICRP report 135 [18], provided an adequate sample is available for accurate evaluation and analysis. As a result, the DRL value that represents our sector will be set at the median distributions of the MGD, i.e., organ dose quantity, obtained from the patient dose registry.

For optimization and auditing purposes, we considered classifying our sample cohort into several CBT groups that encamp the entire CBT range presented for all patients enrolled in this study. DRL values will be established for each mammogram study and for each breast image technique/view. The DRL values established for each breast image view will be classified based on the CBT groups. A minimum of 10 cases per breast image technique/view for each CBT group will be considered and used to calculate the DRL values. No DRL value will be reported for breast image technique/views with less than 10 views.

### Statistical Analysis

GraphPad Prism 8, V8.03, GraphPad Software, La Jolla, CA, USA was used to generate the statistical analysis. Quantitative variables were expressed as median, minimum (Min), maximum (Max) and 5th, 25th, 75th and 95th percentiles. LDRL values based on CBT were established for all possible bilateral breast image views to set as a benchmark and to highlight which breast image view and study yields the highest dose for the patient. Statistical differences between all the possible breast image views were calculated using the Kruskal–Wallis test. Laterality (R and L) statistical difference was calculated using the Mann–Whitney test.

## 3. Results

Depending on the clinical indication and patient’s history, radiologists advise on the type of mammogram study to perform as shown in Table 1.

### 3.1. Scan Acquisition Parameters

All patients enrolled in this study were scanned using a single full-field digital mammography unit that comes with a GEMS amorphous silicon digital detector that has a field of view (FOV) of 24 cm × 30.7 cm and image matrix of 2394 × 3062. This mammogram unit facilitates an automatic exposure control (AEC) mode, as well as an automatic optimization of parameters (AOP). AOP provides the automatic change of the target from Rhodium to Molybdenum and vise versa depending on the X-ray beam spectra needed to perform a breast image view, which depend mainly on the CBT. In association with this, the HVL also changes from 0.4 mm for the target filter combination (Rhodium/Rhodium) to 0.35 mm for Molybdenum/Rhodium. Table 2 offers a descriptive summary of the scan acquisition parameters acquired to perform each breast image technique/view. Scan acquisition parameters are defined per each image view (2D CC/MLO, 3D MLO, 2D SCC/SMLO, 2D MSCC/MSMLO, 2D IMCC/IMMLO, 2D SBCC and 2D CECC/CEMLO). Bilateral spot compression with magnification views were acquired using a 0.1 mm focal spot instead of the 0.3 mm that is used for all other breast image views. Further, bilateral spot compression with magnification views were acquired using a smaller beam collimation.

### 3.2. Patient Characteristic and Diagnostic Mammograms LDRL

**Figure 1:**
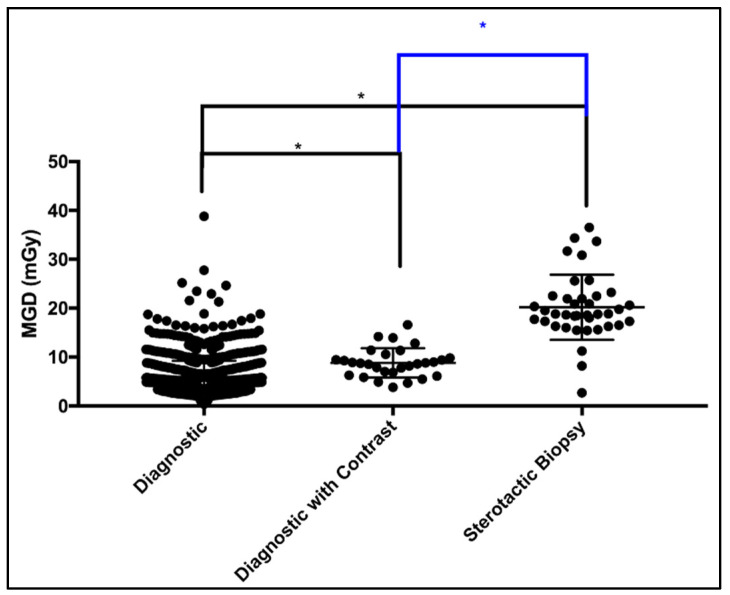
Scatter plot demonstrating the mean and range of MGD per mammogram study offered for diagnosis. * Significant difference, *p* < 0.05.

Patient dose data for a total of 1191 patients from a single healthcare center subjected to (diagnostic (without contrast enhancement) mammogram study: 1123, diagnostic with contract enhancement study: 29 and stereotactic biopsy study: 39) were collected and analyzed. A scatter plot (Figure 1) shows the MGD spectrum for all three mammogram studies performed in our practice.

Using the Kruskal–Wallis test, significant differences were seen between diagnostic mammograms with and without contrast. Further, the differences between diagnostic and stereotactic were also found to be significant. Likewise, the difference between diagnostic with contrast study and stereotactic were found to be significant. Significance is denoted by * in Figure 1. Table 3 presents a descriptive summary of sample size (number of patients enrolled), number of possible views per mammography study and the MGD distribution. Stereotactic biopsy study yields the highest overall MGD, followed by the contrast-enhanced mammogram study. This could be, in part, attributed to the number of images taken for every single case when subjected to stereotactic biopsy.

**Figure 2:**
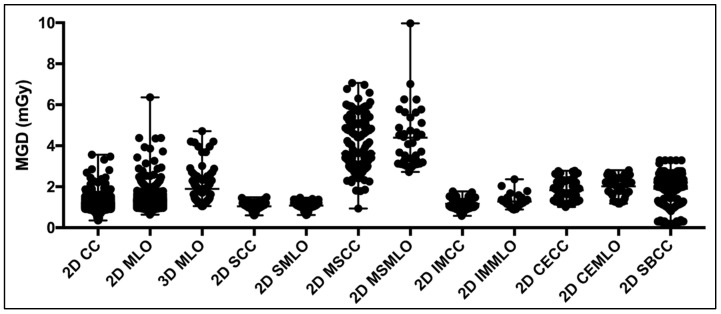
Scatter plot demonstrating mean and range of MGD per all possible image views acquired for diagnostic mammogram.

A scatter plot (Figure 2) shows the MGD spectrum for all possible breast image views performed for all three mammogram studies. Evidently, the 2D spot compression with magnification (2D MSCC/MSMLO) views yield the highest MGD comparing to the rest of the views. This is expected, given the small collimation and focal spot size used to acquire these breast image views. Table 4 demonstrates the MGD distribution in median, 5th, 25th, 75th and 95th percentiles of the entire sample without CBT classification.

**Figure 3:**
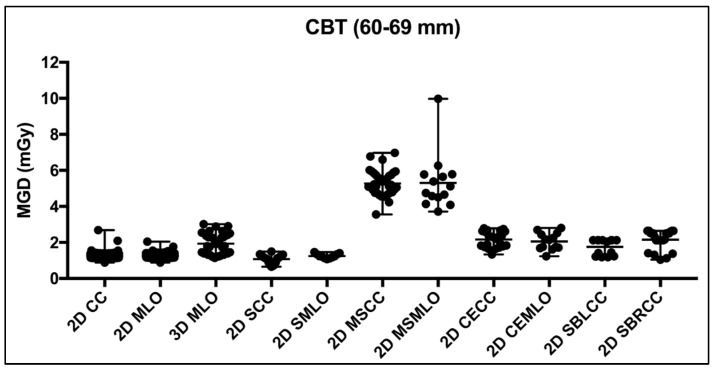
Scatter plot demonstrating the mean and range of MGD for the 6.0–6.9 cm CBT group.

The CBT ranged from 1.9 to 11.0 cm in our total cohort of 1191 patients. Accordingly, nine CBT groups with 10 mm intervals were suggested to assist in dose optimization and internal auditing. To eliminate CBT overlap, the nine CBT groups were classified as <2.0 cm, 2.0–2.9 cm, 3.0–3.9 cm, 4.0–4.9 cm, 5.0–5.9 cm, 6.0–6.9 cm, 7.0–7.9 cm, 8.0–8.9 cm and 9.0–11.0 cm. Other studies like in Switzerland and New South Wales [24] showed results for CBT separated in intervals of 10 mm but with the CBT being grouped as 2.0–3.0, 3.0–4.0 cm etc. Using the Mann–Whitney test, insignificant differences were found between laterality of all views except for the RCC and LCC stereotactic guided biopsy, where a statistical difference of *p* < 0.0001 was seen. Further, as per our practice, the DBT technique is usually performed using the MLO view. Table 5 presents a descriptive summary of all the possible views and the MGD distribution defined for each CBT group. Consequently, all breast image views in Table 5 were demonstrated without laterality except for the SBRCC and SBLCC. Figure 3 shows the mean and range of MGD distribution across the multi-image views for the 60–69 mm CBT group. Statistical difference exists between the MGDs of the different breast image views for the 60–69 mm CBT group. Using the Kruskal–Wallis test, the mean MGD for the standard 2D CC view was found to be significantly different to the mean MGD for the 3D MLO, 2D MSCC, 2D MSMLO, 2D CECC, 2D CEMLO, 2D SBLCC and the 2D SBRCC. Similarly, the mean MGD for the standard 2D MLO was found significantly different to the mean MGD for the 3D MLO, 2D SCC, 2D MSCC, 2D MSMLO, 2D CECC, 2D CEMLO, 2D SBLCC and the 2D SBRCC. The mean MGD for the 3D MLO was found to be significantly different to the mean MGD for the 2D SCC and 2D SMLO using the Kruskal–Wallis test. Further, the mean MGD for the 2D SCC was found to be significantly different to the mean MGD for the 2D MSCC, 2D MSMLO, 2D CECC, 2D CEMLO, 2D SBLCC and 2D SBRCC. Finally, the mean MGD for the 2D SMLO was significantly different to the mean MGD for the 2D MSCC, 2D MSMLO, 2D CECC, 2D CEMLO, 2D SBLCC and 2D SBRCC.

## 4. Discussion

This health sector patient DRL report was made by collecting patients’ measured radiation exposure while undergoing diagnostic mammogram studies in our designated diagnostic mammography healthcare center. Unlike other studies, where DRLs are still reported using PMMA phantoms [25,26], in this patient dose review we were able to document DRLs for all the possible breast image views acquired and techniques used to perform a diagnostic mammogram study in our healthcare sector, including the following: the standard 2D bilateral CC/MLO, the 2D bilateral spot compression CC/MLO, the 2D bilateral spot compression with magnification CC/MLO, the 3D DBT MLO, the 2D bilateral implant CC/MLO, the 2D contrast-enhanced CC/MLO and the 2D bilateral stereotactic guided biopsy CC. Figure 2 illustrates the multi-group comparison, while Table 4 encompasses the descriptive analytics. We established DRL values for every mammogram study using a sufficient sample size (≥10, as demonstrated in Figure 1 and Table 3. Other studies, unlike us, used as low as three images per view to establish DRLs [11]. Here, no DRLs were estimated for views with less than 10 images.

Obvious MGD variations were witnessed across the different possible views, even for the same CBT group. Herein, spot compression with magnification (2D MSCC and 2D SMMLO) views were shown to yield the highest MGD comparing to the rest of the breast image views, with a median MGD of 5.15 and 4.93 mGy, respectively, for the 60–69 mm CBT. In contrast, the median for the other views for the same CBT group ranged from 1.13 to 1.93 mGy, Table 5.

Gennaro et al. [7] reported MGD variations across different centers performing contrast-enhanced diagnostic mammograms. Significant differences were reported using low- and high-energy mammograms; indeed, a high beam energy resulted in a higher MGD.

Regarding the 3D DBT, the median MGD was slightly higher than the 2D standard CC and MLO views but remains in close proximity with the 2D SCC/SMLO, 2D CECC/CEMLO, 2D IMCC/IMMLO and 2D SBCC for the same CBT group. Liu et al. [13] reported a significant difference between the MGD of a standard 2D bilateral CC or MLO and that obtained for the 3D DBT, with the 3D DBT been 1.4 to 2.3 times higher than the standard 2D bilateral views. In contrast, Nicosia et al. [11] studied the MGD across different diagnostic mammogram techniques, including contrast-enhanced mammography, standard bilateral mammography and standard bilateral mammography with one single projection 3D DBT, reporting that the highest MGD seen across the three studies was associated with the standard bilateral one single projection 3D DBT.

Our DRL values for the standard 2D CC and MLO are in line with the reported Switzerland DRLs, specifically for the CBT groups 20–29, 30–39 and 40–49 mm, and we are lower than the reported Switzerland DRLs for the CBT groups 50–59, 60–69, 70–79, 80–89 and 90–110 mm [24].

One need sto acknowledge the different reported statistical quantities in establishing DRLs, especially when considering comparisons alongside other studies. Unlike our use of the median (since the dose review is based on a single center), some studies opt for the 95th percentile and mean values [24,27]. Others, unlike us, do not report the MGD based on CC or MLO views when reporting contrast-enhanced mammogram [9] (Table 4 (CBT not classified) and Table 5 (CBT-classified)).

Limited studies have been published that document dose levels for implant mammography. The existing studies [1,2] have reported on the radiological technique and its impact on image quality, the detectability of features and visualization. To date, no existing study reports DRLs for stereotactic biopsy-guided views. Amir et al. [27] reported an MGD comparison study involving the 3D DBT and stereotactic mammography to guide biopsy. The MGD was reported in mean and standard deviation, i.e., not a DRL. The study concluded that using 3D DBT to guide biopsy would result in a significantly lower MGD compared to the stereotactic guided biopsy, like us. Alcanatara et al. [9] reported the MGD for a stereotactic mammogram without clarifying whether it is a CC or MLO view.

To the authors’ knowledge, this is the first comprehensive patient-based mammography dose report that encompass all possible techniques and image views used to diagnose breast abnormalities. Not only have we established a dose guide for all possible techniques and views, the dose was estimated for several breast thicknesses, as shown in Table 5. In addition, we report the DRL for stereotactic guided biopsy and implant mammography.

The fact that one single designated center and a single digital GE unit were used in this present patient dose review report to generate this DRL system greatly limits its ability to incorporate the influence of different practices and vendors. When considering reporting the DRL for a specific CBT group, we were not able to establish DRLs for all views due to the limited number of images. For example, only the standard 2D CC view was reported for the 20–29 mm CBT group, Table 5. The individual’s breast glandularity was not part of the present report since it is not a readily available variable; likewise with image quality evaluation.

## 5. Conclusions

The present health sector dose report was made using patient dose data and following ICRP report 135. This DRL report can be considered the first thorough diagnostic mammogram report in the Dubai Health sector. DRLs were established for all possible views considered to perform a diagnostic mammogram study. The DRLs reported were based on the two well-known projections (CC and MLO) and eight different CBTs, with 10 mm intervals and zero overlap between CBT groups. The data are representative of the Dubai Health sector in the Emirate of Dubai in the UAE. Spot compression with magnification yielded the highest MGD for a given CBT. The present DRL values are in line with the published DRLs obtained using the same methodology.

## Figures and Tables

**Table 1:** Possible presented clinical indications and the undertaken mammogram study/technique.

Study	Clinical Indication
Diagnostic mammography	Bilateral fibroadenoma follow-up, Breast cancer, Breast implants, Breast assessment, Ductal carcinoma in-situ, Breast lesion, Breast calcification
Magnification technique	Screening, Ductal carcinoma in-situ, Genetic breast cancer risk, Bilateral fibroadenoma
Magnification plus spot technique	Mastectomy, Follow-up, Breast lump, Breast pain
Implant protocol	Breast implants, Implants flipped or punctured, BI-RADS 2, Benign changes, Axillary lump, Follow-up, Breast cancer
Stereotactic biopsy	Guidewire localization stereotactic 1st lesion, Microcalcifications pre-biopsy

**Table 2:** Mammogram acquisition parameters defined per study name and image view.

View (Study Name)	Age (Years)	kVp	mAs	Focal Spot	Collimation
	Min–Max	Min–Max	(Min–Max)		Width/Length
2D CC (Diagnostic)	29–88	25–31	18–251	0.3	24 × 31
2D MLO (Diagnostic)	29–88	26–31	31–303	0.3	24 × 31
3D MLO (Diagnostic)	37–72	26–31	45–297	0.0	24 × 31
2D SCC (Diagnostic)	37–76	25–30	30–72	0.3	13 × 18
2D SMLO (Diagnostic)	40–72	25–30	41–78	0.3	13 × 18
2D MSCC (Diagnostic)	34–84	25–33	14–93	0.1	13 × 18
2D MSMLO (Diagnostic)	32–69	27–33	41–156	0.1	13 × 18
2D IMCC (Diagnostic)	29–68	28–31	56–125	0.3	24 × 31
2D IMMLO (Diagnostic)	29–68	28–31	63–140	0.3	24 × 31
2D CECC (Diagnostic with Contrast)	41–59	28–31	58–135	0.3	24 × 31
2D CEMLO (Diagnostic with Contrast)	41–59	28–31	66–135	0.3	24 × 31
2D SBCC (Stereotactic Biopsy)	35–72	25–31	40–168	0.3	24 × 31

**Table 3:** Mean glandular dose (MGD) Local DRL per study (CBT not classified).

Mammogram	Patients	Number of Images	MGD (mGy)				
			5th per	25th per	Median (DRL)	75th per	95th per
Diagnostic	1123	Minimum 2 up to 10	2.19	4.07	5.06	6.46	12.29
Diagnostic with Contrast	29	Minimum 3 up to 6	4.24	6.57	8.71	10.19	15.41
Stereotactic Biopsy	39	Minimum 10 up to 14	8.18	16.54	18.83	22.49	34.36

**Table 4:** Mean glandular dose (MGD) Local DRL, all possible breast image views (CBT not classified).

View (Study Name)	Number of Images	MGD (mGy)					
		5th per	25th per	Median (DRL)	75th per	95th per	Literature
2D CC (Diagnostic)	1882	0.93	1.07	1.17	1.30	1.50	
2D MLO (Diagnostic)	1820	0.98	1.15	1.27	1.42	1.70	
3D MLO (Diagnostic)	104	1.07	1.28	1.72	2.29	3.96	1.55 * [9]
2D SCC (Diagnostic)	88	0.67	0.95	1.05	1.19	1.39	
2D SMLO (Diagnostic)	49	0.65	0.99	1.08	1.22	1.39	
2D MSCC (Diagnostic)	254	2.48	3.0	3.35	3.96	5.92	
2D MSMLO (Diagnostic)	37	2.94	3.16	4.14	5.25	7.31	
2D IMCC (Diagnostic)	48	0.68	0.96	1.10	1.34	1.66	
2D IMMLO (Diagnostic)	35	0.90	1.07	1.22	1.37	2.10	
2D CECC (Diagnostic with Contrast)	60	2.26	1.33	1.74	2.31	2.74	2.33 *, 2.46 * [7], 1.47 * [9]
2D CEMLO (Diagnostic with Contrast)	61	1.23	1.70	2.14	2.44	2.64	
2D SBCC (Stereotactic Biopsy)	384	0.29	1.77	2.06	2.31	2.66	1.48 * [9]

* Not reported per CC or MLO view.

**Table 5:** Mean glandular dose (MGD) Local DRL per view (CBT-classified).

CBT (mm)	View	Images	MGD (mGy)				
			5th per	25th per	Median (DRL)	75th per	95th per
20–29	2D CC	12	0.66	0.72	0.81	1.14	1.34
30–39	2D CC	67	0.78	0.90	0.97	1.20	1.46
	2D MLO	33	0.80	0.86	0.99	1.19	1.43
	2D SCC	10	0.60	0.88	1.07	1.27	1.39
	2D MSCC	32	2.37	2.56	2.84	3.08	3.63
	2D SBRCC	80	0.94	2.00	2.28	3.08	2.58
	2D SBLCC	54	1.76	1.91	2.04	2.35	2.66
40–49	2D CC	289	0.89	0.96	1.01	1.07	1.17
	2D MLO	133	0.89	0.98	1.03	1.08	1.20
	3D MLO	18	1.05	1.07	1.14	1.27	1.56
	2D SCC	29	0.74	0.95	0.99	1.09	1.20
	2D SMLO	17	0.76	0.93	1.02	1.09	1.21
	2D MSCC	88	2.56	2.92	3.12	3.8	3.60
	2D SBRCC	65	1.24	2.02	2.18	2.59	3.29
	2D SBLCC	57	1.13	2.03	2.06	2.12	2.55
50–59	2D CC	652	0.96	1.06	1.12	1.18	1.27
	2D MLO	399	0.98	1.07	1.14	1.20	1.28
	3D MLO	23	1.14	1.23	1.45	1.92	2.10
	2D SCC	28	0.61	0.95	1.09	1.22	1.40
	2D SMLO	15	0.65	0.97	1.00	1.19	1.24
	2D MSCC	83	2.94	3.22	3.50	3.81	4.13
	2D SBRCC	28	1.02	1.22	2.00	2.76	2.76
	2D SBLCC	12	1.13	1.16	2.01	2.01	2.01
	2D CECC	17	1.01	1.21	1.33	1.73	1.91
60–69	2D CC	569	1.12	1.19	1.25	1.33	1.41
	2D MLO	537	1.13	1.20	1.26	1.33	1.42
	3D MLO	37	1.20	1.37	1.93	2.00	2.91
	2D SCC	18	0.65	0.96	1.13	1.25	1.49
	2D SMLO	11	0.70	1.14	1.23	1.34	1.50
	2D MSCC	36	4.13	4.75	5.15	5.75	6.80
	2D MSMLO	14	3.71	4.12	4.93	5.77	9.97
	2D SBRCC	24	1.06	1.04	2.12	2.34	2.64
	2D SBLCC	14	1.19	1.23	2.09	2.13	2.13
	2D CECC	22	1.38	1.77	2.26	2.62	2.77
	2D CEMLO	13	1.23	1.70	2.14	2.48	1.43
70–79	2D CC	223	1.16	1.31	1.39	1.48	1.66
	2D MLO	467	1.17	1.31	1.40	1.47	1.61
	3D MLO	16	1.39	1.80	2.00	3.45	3.96
	2D IMCC	14	0.58	0.91	1.17	1.47	1.57
	2D IMMLO	10	0.90	0.94	1.26	1.42	1.68
	2D CEMLO	25	1.43	2.15	2.39	2.51	2.63
80–89	2D CC	56	0.92	1.43	1.56	1.77	2.92
	2D MLO	202	1.29	1.47	1.58	1.71	2.22
90–108	2D MLO	41	1.12	1.64	1.88	2.74	4.38

Views with less than 10 images per CBT group were excluded.

## Data Availability

The original contributions presented in this study are included in the article.

## References

[1] Daskalaki A., Bliznakova K., Pallikarakis N. (2016). Evaluation of the effect of silicone breast interts on X-ray mammography and breast tomosynthesis images: A monte Carlo simulation study. Phys. Medica.

[2] Sa dos Reis C., Gremion I., Meystre N.R. (2020). Study of breast implants mammography examinations for identification of suitable image quality criteria. Insights Imaging.

[3] Sharpe R.E., Venkataraman S., Phillips J., Dialani V., Fein-Zachary V.J., Prakash S., Slanetz P.J., Mehta T.S., Østerås B.H., Martinsen A.C.T. (2016). Increased cancer detection rate and variations in the recall rate resulting from implementation of 3D digital breast tomosynthesis into a population-based screening program. Radiology.

[4] Conant E.F., Beaber E.F., Sprague B.L., Herschorn S.D., Weaver D.L., Onega T., Tosteson A.N.A., McCarthy A.M., Poplack S.P., Haas J.S. (2016). Breast cancer screening using tomosynthesis in combination with digital mammography compared to digital mammography alone: A cohort study within the PROSPR consortium. Breast Cancer Res. Treat..

[5] Alakhras M., Bourne R., Rickard M., Ng K.H., Pietrzyk M., Brennan P.C. (2013). Digital tomosynthesis: A new future for breast imaging?. Clin. Radiol..

[6] Asbeutah A.M., AlMajran A.A., Brindhaban A., ASbeutah S.A. (2020). Comparison of radiation doses between diagnostic full-field digital mammography (FFDM) and digital breast tomosynthesis (DBT): A clinical study. J. Med. Radiat. Sci..

[7] Gennaro G., Cozzi A., Schiaffino S., Sardanelli F., Caumo F. (2022). Radiation dose of contrast-enhanced mammography: A two-center prospective comparison. Cancers.

[8] Alcantara R., Posso M., Arenas N., Ejarque B., Lotti V., Besutti G. (2023). Contrast-enhanced mammography-guided biopsy: Technical feasibility and first outcomes. Eur. Radiol..

[9] Alcantara R., Azcona J., Pitarch M., Arenas N., Castells X., Milioni P., Lotti V., Besutti G. (2024). Breast radiation dose with contrast-enhanced mammography-guided biopsy: A retrospective comparison with stereotactic and tomosynthesis guidance. Eur. Radiol..

[10] Jochelson M.S., Lobbes M.B. (2021). Contrast-enhanced mammography: State of the Art. Radiology.

[11] Nicosia L., Bozzini A.C., Pesapane F., Rotili A., Marinucci I., Signorelli G., Frassoni S., Balestreri N., Corso G., Cassano E. (2023). Breast digital tomosynthesis versus contrast-enhanced mammography: Comparison of diagnostic application and radiation dose in screening setting. Cancer.

[12] Hooshmand S., Reed W.M., Suleiman M.E., Brennan P. (2020). Breast-iRRISC: A novel model for predicting the individualized lifetime risk of radiation-induced breast cancer from a single screening event. Br. J Radiol..

[13] Liu J., Zaershenas A., Qadir A., Wei Z., Yang L., Fajardo L., Suzuki K., Qadir A. (2018). Radiation dose reduction in digital breast tomosynthesis (DBT) by means of deep-learning-based supervised image processing. Medical Imaging 2018: Image Processing.

[14] Lee C.S., Aminololama-Shakeri S., Appleton C.M. ACR Practice Parameter for the Performance of Screening and Diagnostic Mammography. Revised 2023 (Resolution 10). https://wiki.radiology.wisc.edu/images/a/a8/SOG_Outreach_ACRattachment.pdf.

[15] Baek J.E., Kang B.J., Kim S.H., Lee H.S. (2017). Radiation dose affected by mammographic composition and breast size: First application of a radiation dose management system for full-field digital mammography in Korean women. World J. Surg. Oncol..

[16] Liu Q., Suleimani M.E., McEntee F.M., Soh B.P. (2020). Diagnostic reference levels in digital mammography: A systematic review. J. Radiol. Prot..

[17] Dalah E.Z., Alkaabi M.K., Ai-Awadhi H.M., Antony N.A. (2024). Screening mammography diagnostic reference level system according to compressed breast thickness: Dubai Health. J. Imaging.

[18] Vano E., Miller D.L., Martin C.J., Rehani M.M., Kang K., Rosenstein M., Ortiz-Lopez P., Mattsson S., Padovani M.R., Rogers A. (2017). Diagnostic reference levels in medical imaging. Ann. ICRP.

[19] ICRP (2007). Radiological protection in Medicine. Annals of the ICRP.

[20] ICRP (2007). The 2007 recommendations of the international commission on radiological protection. Annals of the ICRP.

[21] Hendrick R.E. (2010). Radiation doses and cancer risks from breast imaging studies. Radiology.

[22] BEIR VII Council NR (2006). Health Risks from Exposure to Low Levels of Ionizing Radiation: BEIR VII Phase.

[23] Qaelum N.V. (2019). Dose Patient Radiation Dose Monitoring System User Manual, Version 19.02.

[24] Dupont L., Aberle C., Botsikas D., Ith M., Lima T.V., Menz R., Monnin P., Poletti P.A., Presilla S., Schegerer A. (2024). Proposed DRLs for mammography in Switzerland. J. Radiol. Prot..

[25] Weir A., Schofield K.A., McCurrach A. (2021). Setting Scottish diagnostic reference levels for mammography incorporating both craniocaudal and oblique projections between 30 and 80 mm. J. Radiol. Prot..

[26] Parmaksız A., Ataç G.K., Bulur E., Inal T., Alhan A. (2020). Average glandular doses and national diagnostic reference levels in mammography examinations in Turkey. Radiat. Prot. Dosim..

[27] Amir T., Zuckerman S.P., Barufaldi B., Maidment A.D., Conant E.F. (2021). Comparison of radiation dose between 2D digital stereotactic versus digital breast tomosynthesis-guided breast biopsy. Eur. J. Radiol..

